# Outcome after vaginal delivery of women with a previous medical history of surgically corrected anorectal malformations: a systematic review

**DOI:** 10.1186/s12884-023-05389-9

**Published:** 2023-02-04

**Authors:** Ayla C. de Waal, Tim van Amstel, Judith J. M. L. Dekker, Johannes C. F. Ket, Caroline F. Kuijper, Concetta M. Salvatore, Justin R. de Jong, Ramon R. Gorter

**Affiliations:** 1 Department of Paediatric Surgery, Emma Children’s Hospital, Amsterdam UMC, University of Amsterdam and Vrije Universiteit Amsterdam, P.O. Box 22600, 1100 DD Amsterdam, the Netherlands; 2 Department of Obstetrics and Gynecology Division of Reproductive Medicine, Amsterdam UMC, University of Amsterdam and Vrije Universiteit Amsterdam, Amsterdam, the Netherlands; 3 Medical Library, Vrije Universiteit Amsterdam, Amsterdam, the Netherlands; 4 Department of Paediatric Urology, Emma Children’s Hospital, Amsterdam UMC, University of Amsterdam and Vrije Universiteit Amsterdam, Amsterdam, the Netherlands

**Keywords:** Anal atresia, Anorectal malformation, Cloaca, Delivery, Mode of delivery, Obstetrical complications, Systematic review, Vaginal delivery

## Abstract

**Objective:**

Discussion remains on how to advise women with a past medical history of surgically corrected anorectal malformations (ARMs) regarding vaginal delivery. The aim of this review is to evaluate and review the reported obstetrical complications and outcomes after vaginal delivery for these women.

**Data sources:**

A systematic search was performed from inception up to 25 July 2022 in PubMed, Embase.com and Clarivate Analytics/Web of Science Core Collection, with backward citation tracking.

**Study eligibility criteria/appraisal:**

All articles reported on the outcomes of interest in women with a past medical history of surgically corrected anorectal malformation and had a vaginal delivery were included with the exception of editorial comments or invitational commentaries. Screening, data extraction and risk of bias assessment was done by two authors independently with a third and fourth reviewer in case of disagreement. Tool for Quality assessment depended on the type of article. As low quality evidence was expected no meta-analysis was performed.

**Results:**

Only five of the 2377 articles screened were eligible for inclusion with a total of 13 attempted vaginal deliveries in eight women. In three patients complications were reported: failed vaginal delivery requiring urgent cesarean section in two patients, and vaginal tearing in one patient.

**Conclusion:**

High quality evidence regarding outcomes and complications after vaginal delivery in women with a history of surgically corrected anorectal malformation is lacking. Therefore, based upon this systematic review no formal recommendation can be formulated regarding its safety. Future studies are essential to address this problem.

**Trial registration:**

CRD42020201390. Date: 28–07-2020s.

**Supplementary Information:**

The online version contains supplementary material available at 10.1186/s12884-023-05389-9.

## Background

Anorectal malformations (ARMs) are rare congenital malformations with an estimated incidence of approximately 1 in 5000 live births per year in the Western civilization [1, 2]. The diagnosis is usually made in the early neonatal period and affects both male and female equally [3]. As known, a wide variety of anorectal malformations exist, ranging from perineal fistulas to more complex fistulas, almost all to the urogenital tract. In general, functional outcome is worse in patients with more complex types of anorectal malformation, but it also depends on other factors such as the presence of other associated anomalies in spine, spinal cord and urogenital structures. In females, the following types of anorectal malformation can be encountered: isolated imperforate anus (4.8%), rectovestibular fistula (60.3%), rectoperineal fistula (20.6%) and cloacal anomalies (7.9%) [1, 2, 4, 5]. Cloacal malformations can be subdivided based on the length of common channel, namely < 3 cm and > 3 cm, the longer the common channel the more difficult to correct [3]. In most cases of anorectal malformation, surgical correction is necessary and is usually performed at an early age.

As mentioned above, female patients with anorectal malformation may also suffer from (disease-specific) problems related to the gynecological tract such as congenital anatomical anomalies at birth, sexual/intercourse problems, fertility difficulties and obstetrical complications in later life [6]. In up to 20% of patients with rectovestibular fistula, gynecological abnormalities, such as a vaginal septum, bicornuate uterus or in some rare cases even vaginal agenesis are seen [4]. Not only the disease (anorectal malformation and its associated anomalies) itself, but also the surgery needed to correct the anomalies may affect the gynecological tract. This in turn has consequences later on in life. For example, increased damage can be expected during vaginal delivery in a scarred reconstructed perineum. The anal sphincter and in some women the reconstructed urethra or vagina, may be at risk for dysfunctioning after vaginal delivery, caused from the significant stretch or even ruptures of the perineum [7]. Intensive guidance and counselling regarding pregnancy and mode of delivery is therefore essential for patients with anorectal malformation. It is generally recommended to perform a cesarean section (CS) for all patients with a past medical history of anorectal malformation. Some surgeons however believe that in certain types of anorectal malformation, for instance rectovestibular or rectoperineal fistulas, vaginal delivery could be possible [2]. To our knowledge evidence regarding this topic is scarce, regardless of type of anorectal malformation. As a result, current recommendations seem to be based little and on low-quality evidence (e.g. expert opinions).

## Objective

Therefore, the aim of this systematic review is to determine which obstetrical complications and outcomes have been reported for women with a past medical history of surgically corrected anorectal malformation that gave birth vaginally.

## Materials and Methods

The protocol of this systematic review was registered at PROSPERO: International prospective register of systematic reviews with identification number CRD42020201390. This systematic review was reported according to the Preferred Reporting Items for Systematic Reviews and Meta-Analyses (PRISMA) statement [8]. Although this systematic review was performed due to questions from our patients, they were not actively involved in the design and conduct of this review. Nor did this research receive any funding.

### Eligibility criteria

The eligibility criteria were established using the PICO framework as follows: P(articipants); women with a past medical history of surgically corrected anorectal malformation, I(ntervention); vaginal delivery, C(omparison); no comparison was made, O(utcome); obstetrical complications and outcome [9].

#### Inclusion criteria

Only articles that reported our primary outcome, namely the number of patients with complications after vaginal delivery in women with a past medical history of surgically corrected anorectal malformation, were included in this systematic review. All types of studies are included, with the exception of editorial comments and invited commentaries. Language was restricted to English and Dutch.

#### Exclusion criteria

Articles describing our primary outcome in women with Hirschsprung disease and women with a sphincter rupture in their past medical history (without anorectal malformation) were excluded from this study. In addition, studies describing only the outcomes of cesarean section were also excluded. There were no restrictions in age or type of anorectal malformation.

### Search strategy and information sources

A systematic search was performed (by AW and JCFK) from inception up to 25 July 2022 in PubMed, Embase.com and Clarivate Analytics/Web of Science Core Collection. Keywords (including synonyms and closely related words) were anorectal malformations, cloaca, imperforate anus, natural or vaginal delivery. The full search strategy is shown in Appendix A. To ensure that all possible publications were included, the citations list from all full text screened articles were checked.

### Study selection 

Abstracts were screened independently by two reviewers (AW, TA) according to the in- and exclusion criteria as described above. After which, full text assessment of the selected articles was performed. Disagreements were resolved by consulting a third and fourth reviewer (RG, JD). If studies seemed eligible, but individual data or the primary outcome was lacking/not identified in the study, the first and last author were contacted by mail to obtain more information about their article in order to include them in our systematic review.

### Data extraction

After full-text screening, data extraction was done by two reviewers independently (AW, TA) using a standardized data extraction form. Again, disagreements were resolved by a third and fourth reviewer (RG, JD). The following data were extracted: study design, type of ARM, type of correction for ARM, age at the time of the delivery, gestational age, birth weight, gravida/para, type of delivery, complications, type of pain relief, length of hospital stay and functional outcomes (incontinence, sexual, defecation).

### Outcomes

The primary outcome was the number of patients with complications after vaginal delivery in women with a past medical history of surgically corrected anorectal malformation, as reported by the original paper. Severity of complications were assessed according to the Clavien-Dindo Scale [10, 11]. Secondary outcomes were the type of perineal- and sphincter ruptures as reported by the original paper. Also, other obstetrical outcomes such as defecation problems, sexual problems and urinary problems as reported by the original paper were collected.

### Assessment of risk of bias

Risk of bias was assessed using the appropriate tool according to the type of study, for instance for RCTs we planned to use the Cochrane risk of bias tool [12]. For comparative cohort studies, we anticipated to use the Newcastle–Ottawa scale if possible [13]. As the original New-Castle Ottawa scale is not applicable for case series, it was decided to use the adapted version as described by Hassan Murad et al [14]. Two reviewers (AW, TA) performed the risk of bias analysis.

### Data synthesis

As it was expected to encounter a low number of studies of low quality it is already anticipated to not perform a meta-analysis. Instead only descriptive variables of the included studies will be displayed. Regarding our primary outcome the number of patients are displayed. Results are presented in various tables and figures, as absolute numbers.

## Results

### Study selection

**Fig. 1:**
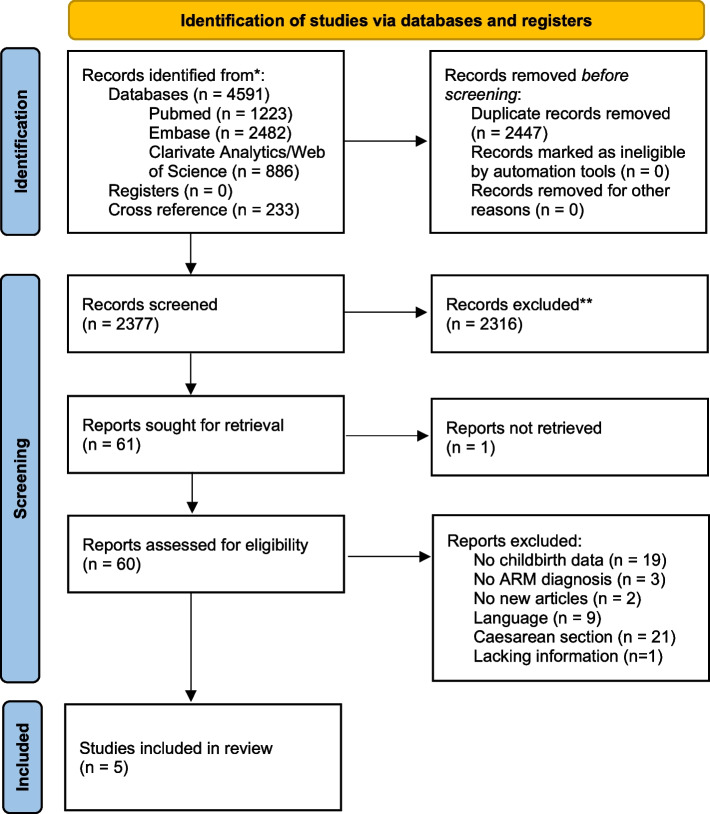
Prisma flowdiagram

The search yielded a total of 4591 articles. After removal of duplicates, 2144 articles were screened by title and abstract. In addition, we screened 233 articles through the citations lists from the concerning articles. In total, 2377 articles were screened. Overall, out of the 60 articles that were assessed by full-text, five articles were eligible for inclusion (Fig. 1).

### Study Characteristics

#### Included studies

The general characteristics of the included studies are shown in Table 1 [15–19]. In the two retrospective case series, complications during vaginal delivery were not specifically mentioned [16, 17]. Because of this, contact was made, as mentioned above, with Peña et al. who reported that no complications had occurred [17]. Unfortunately, we did not receive an answer from Iwai et al. where they stated that all women had a normal vaginal delivery [16]. Therefore, the assumption was made that these women did not experience any complications during delivery. For these reasons, both articles were included. Appiah-Sakyi et al. reported a woman who failed to deliver vaginally after an hour of pushing, after which a cesarean section was performed [18]. They discovered that she had a blind-ending pouch of her uterine cavity that had no connection with the cervical canal. Because of missing data regarding the anatomical description and how she got pregnant, contact was made with the authors [18]. Unfortunately, we did not receive an answer. Thereby the assumption was made that she had a non-communicating rudimentary horn with cavum, in which a vaginal delivery was never an option. Kawaguchi et al. reported a woman who progressed to complete dilation, but experienced arrest of descent after 3,5 h in the second stage of labor [19]. An emergency cesarean section was performed [19].

**Table 1:** General characteristics of the included studies [15–19]

	**Study design**	**Number of patients**	**Type of ARM**	**Type of correction**	**Age, years**	**Obstetrical history**	**Type of delivery**
Greenberg et al. 1997 [15]	Case report	1	Cloaca	Colostoma, Rectal pull through procedure, repair of rectovaginal fistula	27	Gravida1, Para 0	Vaginal VAVD FAVD
Iwai et al. 2007 [16]	Retrospective case series	3	#1. High type ARM #2. Low type ARM #3. Low type ARM	#1. Colostomy + abdominoperineal rectoplasty #2 and #3. Neonatal perineoplasty	#1. 31 #2. 40 #3. 25	#1. Gravida unknown, Para 2 #2. Gravida unknown, Para 5 #3. Gravida unknown, Para 1	Vaginal
Peña et al. 2004 [17]	Retrospective case series	2	Cloaca with common channel < 3 cm	Posterior sagittal approach with total urogenital mobilization (TUM)	N/R	N/R	Vaginal
Appiah-Sakyi et al. 2009 [18]	Case report	1	Imperforate anus	N/R	25	Gravida 1, Para 0	Cesarean section
Kawaguchi et al. 2021 [19]	Case report	1	Cloaca	Posterior sagittal anorectoplasty	18	Gravida 1, Para 0	Cesarean section

*N/R* not reported in original article, *VAVD* vacuum-assisted vaginal delivery, *FAVD* forceps-assisted vaginal delivery

Furthermore, due to the missing data there is a large heterogeneity among the articles.

#### Excluded studies

From the 60 articles that were screened full-text, 55 articles were excluded. Reasons for exclusion can be found in Fig. 1 and Appendix B. Three articles required additional investigation as there was disagreement between the first two reviewers requiring assessment by the third and fourth reviewer.

Vilanova-Sanchez et al., a systematic review, included 13 articles about obstetrical outcomes in women with a past medical history of an anorectal malformation [6]. In total 24 pregnancies were reported [6]. Two articles included both one patient having a vaginal delivery [6, 15, 20]. One was already included in our review, the other one did not meet the criteria of surgery in the past medical history [15, 20].

Greenberg et al. 2003, a case report, describes the same patient described in the article of 1997 which was included in our review [15, 21]. This second report describes her second pregnancy, after which she delivered through a cesarean Sect.  [21].

Finally, contact was made with the authors of Davies et al. because of lacking information [22]. Unfortunately, the needed information was not available, so no assumptions could be made. Therefore, the article was excluded.

### Risk of bias of included studies

In this review, three case reports and two case series were included [15–19]. As the original New-Castle Ottawa scale is not applicable for case series, it was decided to use the adapted version as described by Hassan Murad et al [14]. The two case series scored poorly, focusing on selection bias and poor description of method. Looking at all the evidence we gathered, we state that level four (poor quality) evidence according to the Oxford Centre for Evidence-Based Medicine for our primary outcome is present [23]. There were no RCT’s so the Cochrane risk of bias tool was not used.

### Synthesis of results

Baseline characteristics of the included studies are displayed in Table 1 [15–19]. Gestational age of the babies was reported by Greenberg et al., Appiah-Sakyi et al. and Kawaguchi et al. and were respectively 34.5 weeks, 32 weeks and 37 weeks [15, 18, 19]. Greenberg et al. and Kawaguchi et al. reported the birth weight, respectively 2310 g and 2428 g [15, 19]. Greenberg et al. reported also type of pain relief, namely an epidural [15]. Length of hospital stay was not reported in any of the studies.

### Primary outcome

Our primary outcome is displayed in Table 2. In total we included in this systematic review 13 attempted vaginal deliveries in eight women. Of these attempts, two patients required a cesarean section as vaginal delivery failed. Due to the lack of information on the anesthesia techniques, this complication was scaled as Clavien-Dindo IIIA/IIIB [18, 19]. In another woman two vaginal tears occurred (one large left vaginal sulcus tear (Grade IIIA) and small midline introital tear (Grade IIIA)) after Tucker-McLean Forceps were applied [15]. Therefore four complications occurred in three women.

**Table 2:** Overview primary outcome in the included studies [15–18]

	**Number of patients**	**Total number of successful vaginal deliveries**	**Total number of complications**	**Clavien-Dindo classification**
Greenberg et al. 1997 [15]	1	1/1	2	Grade IIIA
Iwai et al. 2007 [16]	3	8/8	0	N/A
Peña et al. 2004 [17]	2	2/2	0	N/A
Appiah-Sakyi et al. 2009 [18]	1	0/1	1	Grade IIIA or Grade IIIB
Kawaguchi et al. 2021 [19]	1	0/1	1	Grade IIIA or Grade IIIB

*N/A* not applicable, *N/R* not reported

### Secondary outcomes

Table 3 displays the secondary outcomes for each study. Functional outcome was reported in three studies [15, 18, 19]. Greenberg et al. reported no defection problems, Appiah et al. reported no urinary or fecal incontinence and Kawaguchi et al. reported urinary retention which required intermittent self-catheterization for three months [15, 18, 19]. However, it is unclear what kind of follow-up was done. Sexual problems were not mentioned in the articles.

**Table 3:** Overview of the specific obstetrical complications in the included studies [15–19]

	**Complications**	**Clavien-Dindo classification**	**Functional outcome**
Greenberg et al. 1997 [15]	#1. Large left vaginal sulcus tear #2. Small midline introital tear	#1. Grade IIIA #2. Grade IIIA	Well healed. Complete return of baseline bowel function
Iwai et al. 2007 [16]	No complications	N/A	N/R
Peña et al. 2004 [17]	No complications	N/A	N/R
Appiah-Sakyi et al. 2009 [18]	Cesarean section	Grade IIIA or Grade IIIB	No residual urinary or fecal incontinence
Kawaguchi et al. 2021 [19]	Cesarean section	N/A	Urinary retention requiring intermittent self-catheterization for three months

*N/A* not applicable, *N/R* not reported in original article

## Discussion

### Principal findings

This systematic review shows that literature regarding obstetrical outcomes and complications after vaginal delivery in women with a medical history of surgically corrected anorectal malformation is scarce and of low quality. Formal recommendation on the mode of delivery can therefore not be made. Paucity of the literature indicates the necessity of larger studies investigating the obstetrical outcomes and complications in women with surgically corrected anorectal malformation.

### Comparison with existing literature

To our knowledge, this is the first systematic review performed according to the PRISMA methodology regarding this subject. It provides new information that can be used to counsel pregnant patients with a medical history of surgically corrected anorectal malformation. A recent literature study on this matter concluded that a cesarean section is preferable in patients with a cloacal repair, as these patients all have undergone some type of extensive correction of the perineal body and vagina [6]. It is assumed that these patients have an increased risk of damage to these structures during vaginal delivery because scar tissue does not stretch as well as healthy tissue. The authors based their conclusion on their review comprising 13 different studies. However, only two studies reported results of vaginal delivery [6]. In line with our finding, they also state that there is a paucity of evidence-based data.

In our systematic review only five articles were eligible for inclusion. All studies were of low-quality due to the study design with consequent methodological flaws. As our interest was vaginal delivery in patients with a corrected ARM we did not report the outcomes after a cesarean section which led to the exclusion of 21 articles. Only Appiah et al. and Kawaguchi et al. were included because vaginal delivery was attempted before performing a cesarean section.  [18, 19]. Appiah et al. shows the importance of screening for gynecological malformations in female patients with a history of anorectal malformation: anatomical abnormalities may be present and might affect the choice of delivery method [18, 24]. Therefore, screening for gynecological malformations, e.g. with ultrasound, is recommended in patients with an ARM in their medical history. Collaboration between pediatric surgery and gynecology is essential in order to deliver optimal care in these patients [24].

Most clinicians consider cloacal malformations as complex anorectal malformations requiring extensive surgery at young age. These patients might be prone to damage to their birth canal and pelvic floor by extensive stretching during vaginal delivery possibly resulting in ruptures. Therefore, in most patients, a cesarean section is advised. In other types of anorectal malformations recommendations regarding mode of delivery are not specifically made. Another possible reason for the relatively high number of cesarean sections in patients with a history of ARM may be due to cultural differences. In general, more cesarean sections are conducted in Latin America and the Caribbean region [25].

Currently, the decision to perform a cesarean section in our population is based on expert opinion, the severity of the condition of the regarding patient and the experience of the obstetrician and pediatric surgeon. Any consensus based on the current literature is lacking at the time of writing this study.

One must bear in mind that a cesarean section can also be a potential harmful procedure for both mother and child. For example, the incidence of postoperative ileus after cesarean section is approximately 12% [26]. In addition, there is a higher risk of postpartum sepsis and subsequent admittance to the ICU, especially in case of an emergency cesarean section.  [25, 26]. Subsequent cesarean sections and an uterus rupture in the medical history can result in even higher risks. Kramer et al. found a 47% increase in abnormal placentation and a 40% increase in placental abruption [27]. The number of placenta accretes directly correlates with the number of previous cesarean sections.  [27]. For 1–5 cesarean sections in the past medical history the percentages are respectively 3%, 11%, 40%, 61% and 67.1% [27]. Patients with a surgically corrected cloaca most likely underwent additional procedures like a bladder augmentation [6]. These procedures address caution when performing a cesarean section due to the risk of iatrogenic damage. However, the risk of bleeding in a planned cesarean section is lower in comparison to a planned vaginal birth (respectively 1.1% and 6.0%) [26].

Apart from adverse effects on the mother, a cesarean section can have disadvantages for the child as well. The lungs of a newborn should be cleared rapidly to allow gas exchange for a smooth transition to air breathing in order to prevent respiratory morbidity. Tefera et al. performed a systematic review and meta-analysis on the risk of neonatal respiratory morbidity in elective cesarean section vs vaginal delivery. Children born by elective cesarean section experienced significantly more respiratory problems compared to vaginal delivery [28].

As a result of the mentioned arguments a vaginal delivery is preferred over a cesarean section in the Netherlands. A thorough risk assessment must be performed considering the wellbeing of both mother and child. This issue deserves further international attention, particularly for women with a history of ARM.

Approximately 2.4% of healthy women will develop obstetrical anal sphincter injuries (OASI) as a result of vaginal delivery [29]. Recent studies show that tearing of the perineum is a risk factor for developing urinary incontinence, fecal incontinence and dyspareunia [29, 30]. Although patients are at risk for these complications and the numbers are low, Iwai et al. and Peña et al. showed that a vaginal delivery is possible without complications in patients with an anorectal malformation. Additional research is needed to provide a recommendation about the mode of delivery in pregnant women with a history of anorectal malformations.

### Strengths and limitations

This systematic review included five studies of poor quality mainly due to the methodology (i.e. case series). Large heterogeneity in these series therefore existed regarding patient selection and outcome definitions. Although in most studies general statements regarding complications of vaginal delivery were made, only two studies explicitly described them [15, 18]. The secondary outcomes of this study were absent in most of these studies and therefore no conclusions could be drawn regarding this subject. Secondly, due to the small sample size and poor-quality data, no general recommendation can be made. As mentioned above selection bias is present in most studies included in this study. In many cases it was unclear why the decision was made to deliver vaginally. Additionally, selection bias/indication bias due to cultural differences as stated above could be of importance. Although we performed an extensive literature search, it was decided not to search for unpublished data or grey literature. Therefore, we could have missed some articles. Lastly, pilot was conducted to ensure inter-rater reliability.

## Conclusion and implications

In conclusion, high quality evidence regarding obstetrical outcomes and complications after vaginal delivery in women with a medical history of anorectal malformation is highly scarce in the current literature. Therefore, no recommendation can be made. Additional large studies are needed to investigate the obstetrical outcomes and complications in women with surgically corrected anorectal malformation. Furthermore, the development of a core outcome set in this specific patient group should be developed.

## Supplementary Information

**Additional file** **1.**

**Additional file** **2.**

## Data Availability

Not applicable.

## Abbreviations

ARM: Anorectal malformations
CS: Cesarean section
OASI: Obstetrical anal sphincter injuries
