# Supplementary material for: Outcome after vaginal delivery of women with a previous medical history of surgically corrected anorectal malformations: a systematic review
Source: BMC Pregnancy Childbirth. 2023 Feb 4;23:94. doi: 10.1186/s12884-023-05389-9 (PMC9898899; doi:10.1186/s12884-023-05389-9)
Supplement: Supplementary file 1 — Additional file 1. [file 12884_2023_5389_MOESM1_ESM.docx]

**Additional file 1 – appendix A**

**Search strategy for PubMed (25 July 2022)**

| **Search** | **Query** | **Results** |
| --- | --- | --- |
| **#3** | **#1 AND #2** | **790** |
| **#2** | **(("Delivery, Obstetric"[Mesh] OR "Parturition"[Mesh] OR "parturi*"[tiab] OR "birth*"[tiab] OR "childbirth*"[tiab] OR "deliver*"[tiab] OR "labor*"[tiab] OR "labour*"[tiab] OR "obstetr*"[tiab]) AND ("Vagina"[Mesh] OR "vagina*"[tiab])) OR "Cesarean Section"[Mesh] OR "cesarea*"[tiab] OR "caesarea*"[tiab] OR "c section"[tiab] OR "c sections"[tiab] OR ("abdominal"[tiab] AND "deliver*"[tiab]) OR "postcesarea*"[tiab] OR "postcaesarea*"[tiab] OR "Pregnancy Outcome"[Mesh] OR "pregnancy outcome*"[tiab] OR "birth outcome*"[tiab]** | **200,287** |
| **#1** | **"Anorectal Malformations"[Mesh] OR "Rectal Fistula"[Mesh] OR "Anus, Imperforate"[Mesh] OR (("Anus Diseases"[Mesh:NoExp] OR "Anal Canal"[Mesh] OR "Rectum"[Mesh] OR "anorectal*"[tiab] OR "rectovestibula*"[tiab] OR "rectoperineal*"[tiab] OR "cloaca*"[tiab] OR "rectovagina*"[tiab] OR "rectal*"[tiab] OR "recto"[tiab] OR "rectum"[tiab] OR "anus"[tiab] OR "perianal*"[tiab] OR "rectoanal*"[tiab] OR "anal"[tiab]) AND ("Congenital Abnormalities"[Mesh:NoExp] OR "malformati*"[tiab] OR "abnormal*"[tiab] OR "deformati*"[tiab] OR "atresi*"[tiab] OR "anomal*"[tiab] OR "fistul*"[tiab] OR "imperforat*"[tiab]))** | **25,190** |

**Search strategy for Embase.com (25 July 2022)**

| **No.** | **Query** | **Results** |
| --- | --- | --- |
| **#3** | #1 AND #2 | **1,678** |
| **#2** | 'vaginal delivery'/exp OR (('obstetric delivery'/de OR 'birth'/de OR parturi*:ti,ab,kw OR birth*:ti,ab,kw OR childbirth*:ti,ab,kw OR deliver*:ti,ab,kw OR labor*:ti,ab,kw OR labour*:ti,ab,kw OR obstetr*:ti,ab,kw) AND ('vagina'/exp OR vagina*:ti,ab,kw)) OR 'cesarean section'/exp OR cesarea*:ti,ab,kw OR caesarea*:ti,ab,kw OR 'c section':ti,ab,kw OR 'c sections':ti,ab,kw OR ((abdominal NEAR/3 deliver*):ti,ab,kw) OR postcesarea*:ti,ab,kw OR postcaesarea*:ti,ab,kw OR 'pregnancy outcome'/exp OR 'pregnancy outcome*':ti,ab,kw OR 'birth outcome*':ti,ab,kw | **259,681** |
| **#1** | 'anorectal malformation'/exp OR 'rectum fistula'/exp OR (('anus disease'/de OR 'anus sphincter disorder'/exp OR 'rectum disease'/de OR 'anorectal disease'/exp OR 'rectovaginal fistula'/exp OR 'rectum injury'/exp OR 'rectum perforation'/exp OR anorectal*:ti,ab,kw OR rectovestibula*:ti,ab,kw OR rectoperineal*:ti,ab,kw OR cloaca*:ti,ab,kw OR rectovagina*:ti,ab,kw OR rectal*:ti,ab,kw OR recto:ti,ab,kw OR rectum:ti,ab,kw OR anus:ti,ab,kw OR perianal*:ti,ab,kw OR rectoanal*:ti,ab,kw OR anal:ti,ab,kw) AND ('congenital disorder'/de OR 'congenital malformation'/de OR 'newborn disease'/de OR malformati*:ti,ab,kw OR abnormal*:ti,ab,kw OR deformati*:ti,ab,kw OR atresi*:ti,ab,kw OR anomal*:ti,ab,kw OR fistul*:ti,ab,kw OR imperforat*:ti,ab,kw)) | **39,552** |

**Search strategy for Clarivate Analytics/Web of Science Core Collection (25 July 2022)**

| **#1** | TS=((“anorectal*” OR “rectovestibula*” OR “rectoperineal*” OR “cloaca*” OR “rectovagina*” OR “rectal*” OR “recto” OR “rectum” OR “anus” OR “perianal*” OR “rectoanal*” OR “anal”) AND (“malformati*” OR “abnormal*” OR “deformati*” OR “atresi*” OR “anomal*” OR “fistul*” OR “imperforat*”) AND (((“parturi*” OR “birth*” OR “childbirth*” OR “deliver*” OR “labor*” OR “labour*” OR “obstetr*”) AND (“vagina*”)) OR (“cesarea*” OR “caesarea*” OR “c section” OR “c sections” OR (“abdominal” NEAR/3 “deliver*”) OR “postcesarea*” OR “postcaesarea*” OR “pregnancy outcome*” OR “birth outcome*”))) | **620** |
| --- | --- | --- |
