# Supplementary material for: Outcome after vaginal delivery of women with a previous medical history of surgically corrected anorectal malformations: a systematic review
Source: BMC Pregnancy Childbirth. 2023 Feb 4;23:94. doi: 10.1186/s12884-023-05389-9 (PMC9898899; doi:10.1186/s12884-023-05389-9)
Supplement: Supplementary file 2 — Additional file 2. [file 12884_2023_5389_MOESM2_ESM.docx]

**Additional file 2 - appendix B: excluded studies and reason for exclusion.**

| **Author/year** | **Study design** | **Number of patients** | **Reason exclusion** |
| --- | --- | --- | --- |
| Acker et al. 2019 | Retrospective cohort | 69 | No childbirth data |
| Adler et al. 2011 | Audit review | 180 | No childbirth data |
| Barg et al. 2021 | Retrospective cohort | 332 | No ARM diagnosis |
| Becker et al. 2009 | Casereport | 1 | Caesarean section |
| Bellmann et al. 1979 | - | - | Language |
| Besnard et al. 2009 | - | - | Language |
| Breech, L. 2010 | Narrative review | N/A | No childbirth data |
| Cardamone et al. 2015 | Literature review | N/A | No childbirth data |
| Chandran et al. 2021 | Casereport | 1 | Caesarean section |
| Couchman et al. 2015 | Retrospective cohort | 19 | No childbirth data |
| Davies et al. 2010 | Retrospective cohort | 44 | Lacking information |
| Dewberry et al. 2020 | Review | N/A | No childbirth data |
| Doga et al. 2015 | Casereport | 1 | No childbirth data |
| Fernando et al. 2015 | Review | N/A | No childbirth data |
| Garalejic et al. 2010 | Casereport | 1 | Language |
| Gezer et al. 2011 | Letter to the editor | 1 | Caesarean section |
| Greenberg et al. 2003 | Casereport | 1 | Caesarean section |
| Greenwell et al. 2003 | Retrospective cohort | 20 | No childbirth data |
| Harris et al. 2021 | Survey | - | No childbirth data |
| Hendren W.H. 1988 | Retrospective cohort | 154 | No childbirth data |
| Iakutina, M. F. 1966 | - | - | Language |
| Joó et al. 2004 | Casereport | 1 | Language |
| Kenevan et al. 2019 | Casereport | 1 | Caesarean section |
| Kobayashi et al. 2021 | Prospective cohort | - | No childbirth data |
| Kubota et al. 2019 | Guideline | N/A | No childbirth data |
| Lewis et al. 2017 | Casereport | 1 | Caesarean section |
| Libby et al. 2017 | Casereport | 1 | No childbirth data |
| Ljubic et al. 1993 | Casereport | 1 | Language |
| Mariona et al. 1982 | Casereport | 1 | Caesarean section |
| Maruotti et al. 2004 | Casereport | 1 | Language |
| McKenzie et al. 2016 | Casereport | 1 | Caesarean section |
| Mincher et al. 2007 | Casereport | 1 | Caesarean section |
| Nezarati et al. 1999 | Casereport | 1 | Caesarean section |
| Paola et al. 2022 | Casereport | 1 | No ARM diagnosis |
| Ragab et al. 2009 | Casereport | 1 | Caesarean section |
| Ramos et al. 2018 | - | - | Language |
| Resnik et al. 1992 | Casereport | 1 | Caesarean section |
| Rintala, R.J. 2015 | Literature review | N/A | No childbirth data |
| Rochet et al. 1980 | - | - | Language |
| Rotem et al. 2021 | Retrospective cohort | 332 | No ARM diagnosis |
| Roziana et al. 2021 | Casereport | 2 | Caesarean section |
| Salvi et al. 2008 | Casereport | 1 | Caesarean section |
| Sattari et al. 2018 | Casereport | 1 | Caesarean section |
| Shamsudin et al. 2013 | Casereport | 1 | Caesarean section |
| Shrim et al. 2008 | Casereport | 1 | Caesarean section |
| Spieth et al. 2020 | Retrospective cohort | 41 | No childbirth data |
| Takashi et al. 2021 | Casereport | 1 | Caesarean section |
| Vaidya, A. S. 2014 | Review | N/A | No childbirth data |
| Van der Heijden et al. 2009 | Casereport | 1 | Caesarean section |
| Versteegh et al. 2021 | Review | N/A | No new articles |
| Vilanova et al. 2017 | Retrospective cohort | 15 | No childbirth data |
| Vilanova et al. 2019 | Systematic review | 16 | No new articles |
| Warman et al. 2011 | Casereport | 1 | Caesarean section |
| Waters, E. G. 1982 | Casereport | 1 | Caesarean section |
| Whitehouse et al. 1949 | Casereport | 1 | No article available |
| Zarate et al. 2015 | Letter to the editor | 1 | No childbirth data |
